# Endoscopic management of acute sigmoid volvulus in high risk surgical elderly patients: a randomized controlled trial

**DOI:** 10.1007/s00423-023-03071-4

**Published:** 2023-08-28

**Authors:** Said Negm, Ahmed Farag, Ahmed Shafiq, Adel Moursi, Amr A. Abdelghani

**Affiliations:** Faculty of Medicine, Zagazig University, Zagazig, Egypt

**Keywords:** Sigmoid volvulus, Endoscopy, Intestinal obstruction, Loop colostomy, Sigmoid fixation, Sigmoid excision

## Abstract

**Background:**

Most patients with sigmoid volvulus are of old age with multiple comorbidities. So, the risk of surgery for those elderly patients is usually associated with increased rates of morbidity and mortality. Early intervention is required for managing sigmoid volvulus to avoid its serious complications; therefore, early endoscopic untwist of sigmoid colon can be performed followed by endoscopic fixation of sigmoid colon under sedation in this category of the patients to avoid development of high risk surgical complications following surgical fixation of sigmoid colon or sigmoidectomy after initial simple loop colostomy procedure to relieve obstruction.

**Methods:**

This prospective randomized controlled clinical trial included all patients who developed acute sigmoid volvulus and were referred to the Zagazig University Hospital Emergency Department between December 2020 and August 2022. The study was prospectively approved by Zagazig University Faculty of Medicine Institutional Review Board (Approval Number: 9989/23-10-2022) and was retrospectively submitted in http://clinicaltrials.gov in November 2022 (http://clinicaltrials.gov ID: NCT05620446). Included eligible patients were simply randomized at a 1:1 ratio to “Endoscopic Group (EG)” or “Surgical Group (SG)” via drawing of sealed envelopes containing computer-generated random numbers prepared by a third party before start of intervention.

**Results:**

Sample size included 18 patients divided into 2 equal groups. (1) Endoscopic group included 9 patients who were subjected to endoscopic untwist of sigmoid colon followed by endoscopic fixation of sigmoid colon under sedation; (2) Surgical group included 9 patients who were subjected to surgical fixation of sigmoid colon or sigmoidectomy after initial simple loop colostomy under general anesthesia. In comparison between both groups, there were statistically significant differences regarding length of hospital stay and procedure time. Unfortunately, there were no statistically significant differences regarding postoperative complications and co-morbidities. Eight patients in the endoscopy group demonstrated excellent quality of life, and one demonstrated good quality of life; unlike the surgical group, there were 3 patients with excellent quality of life, 5 patients with good quality of life, and 1 patient with poor quality of life. So there was statistically significant difference regarding quality of life between both groups. During the 9-month follow-up period, both groups demonstrated no cases of recurrence post-fixation.

**Conclusion:**

Endoscopic management of acute sigmoid volvulus is effective and safe in elderly high risk surgical patients (either in managing the intestinal obstruction caused by volvulus or in definitive treatment of volvulus).

## Introduction

Sigmoid volvulus is a surgical emergency and if left untreated, it results in bowel ischemia and perforation with high mortality rates [1]. Moreover, surgical management of sigmoid volvulus may be associated with increased rates of morbidities and mortalities, particularly in elderly patients with multiple comorbidities [2]. In order to decrease serious complications in previously mentioned group of patients, the earliest and least invasive interventions should be utilized [3]. The deflating rectal tube procedure was performed to untwist sigmoid volvulus; however, if it fails, two-step surgery should be performed: (a) emergency surgical simple loop colostomy to relieve intestinal obstruction; (b) definitive surgery to restore colonic continuity [4]. The minimally invasive “endoscopic approach” has become first line of management to relieve obstruction in uncomplicated sigmoid volvulus in elderly patients [5]. The endoscopic untwist of acute sigmoid volvulus was first described by Bruusgaard, and it has both diagnostic and therapeutic advantages [6]. The diagnostic advantages include (a) evaluation of mucosa and its viability; (b) determining site of the twist (appears spiral and sphincter-like area of the mucosa); (c) exclusion of other causes of intestinal obstruction [7]. The endoscopic approach has reported 75–95% success rate in treating acute sigmoid volvulus; however, recurrence rate may reach up to 90% [8, 9]. To prevent recurrence, a few days later, elective definitive surgery should follow the endoscopic untwist. Due to general condition of the elderly high risk surgical patients, definitive surgery may be postponed. Therefore, in our study, we will try to anchor and tether the sigmoid colon to anterior abdominal wall using the endoscopic approach without need for general anesthesia or major surgery.

### Objectives

To compare effectiveness and safety of endoscopic “anchoring” techniques versus surgical management of acute sigmoid volvulus in high-risk elderly surgical patients with comorbidities.

## Patients and methods

### Patients

This prospective randomized controlled clinical trial included all patients who developed acute sigmoid volvulus and were referred to the Zagazig University Hospital Emergency Department between December 2020 and August 2022. The study was prospectively approved by Zagazig University Faculty of Medicine Institutional Review Board (Approval Number: 9989/23-10-2022) and was retrospectively submitted in clinicaltrials.gov in November 2022 (ClinicalTrials.gov ID: NCT05620446). The study was conducted under the code of ethics of the World Medical Association (Declaration of Helsinki) for studies involving human subjects. Written informed consent was obtained from all participants. This study has been reported in line with Consolidated Standards of Reporting Trials (CONSORT) guidelines.

We included patients aged ≥65 years-old, patients with no prior management by rectal deflating tube, patients with no evidence of bowel ischemia, patients with body mass index <35 Kg/m^2^ and patients of American Society of Anesthesiologists III and IV classes and not in septic shock. Patients who were in good general condition (American Society of Anesthesiologists I and II patients); aged <65 years old; patients with suspected bowel ischemia (patients with suspected bowel ischemia were identified: (a) clinically, by classic triad of abdominal pain, hematochezia and fever in combination with non-specific symptoms like vomiting, bloating, diarrhea, frequent, and forceful bowel movements at first then dead silent abdomen and mental confusion; (b) by laboratory investigations, as marked elevation in WBC, increased serum lactate, metabolic acidosis, increased c-reactive protein and elevated d-dimer; and (c) radiologically, by CT abdomen and pelvis with contrast. Also, endoscopy is diagnostic for colonic ischemia); patients with body mass index > 35 Kg/m^2^ as the higher body mass index is usually associated with failure of endoscopic fixation because of thick and fatty anterior abdominal wall that makes it difficult for two-shot anchor device to reach sigmoid colon lumen, so the thinner the patient, the higher success rate, and patients who were managed by rectal deflating tube were all excluded.

Included eligible patients were simply randomized at a 1:1 ratio to “Endoscopic Group (EG)” or “Surgical Group (SG)” via drawing of sealed envelopes containing computer-generated random numbers prepared by a third party before the start of the intervention.

The sample size was calculated by using an open Epi program depending on the following data; confidence interval 95%, power of the test 80%, ratio of unexposed/ exposed 1, the success rate of surgical sigmoid fixation versus endoscopic fixation was 90% versus 15% respectively [10]. Odd ratio 0.02, and risk ratio 0.17, so the calculated sample size equal 18 patients divided into two equal groups. Primary outcome was management of the intestinal obstruction caused by sigmoid volvulus and long term outcome (secondary) was preventing recurrence of volvulus during the 9-months follow-up period.

### Diagnosis

After full history taking and complete physical examination, acute sigmoid volvulus was clinically suspected and then confirmed by laboratory investigations (complete blood picture, liver and kidney functions, coagulation profile), radiological imaging ( X-ray of the chest and abdomen, abdominal US, CT abdomen with oral and I V. contrast and virtual colonoscopy).

### Intervention

In this study, the investigators tried to relieve intestinal obstruction due to sigmoid volvulus then preventing recurrence of volvulus only in high risk surgical elderly patients with high risk of anesthesia. Initially, a rectal deflating tube was tried in all patients with a high success rate in relieving obstruction but recurrence was the rule and a conflicting issue especially in this category of patients. Thus, in this study, the investigators focused only on this category of the patients who were not responding to the rectal deflating tube. Patients who were managed by rectal deflating tube were excluded from the start of the trial. Therefore, randomization of the patients was only done after rectal deflating tube trial failure. Patients in endoscopic group were subjected for endoscopic untwist of volvulus first then endoscopic fixation of sigmoid colon, while patients in surgical group were subjected for simple loop proximal colostomy first then sigmoid fixation or excision.

For Endoscopic Group: no colostomy was performed, but it was just a transmural fixation. After admission of patients to the emergency department and confirmation of the diagnosis, the included patients in this group were subjected for rapid resuscitation in ICU (intensive care unit) or inpatient bed according to patient’s general condition. The resuscitated patients rapidly underwent colonoscopy under sedation in operative room (OR) to untwist the volvulus by passing the colonoscopy (CF260 series; Olympus, Tokyo, Japan) to the site of volvulus. The untwist was performed under radiological guidance of C-arm by gas suction and insufflation till untwisting was achieved. Then the rectal tube was inserted under colonoscopic guidance and visualization to avoid recurrence of volvulus and also for bedside washing and irrigation of the colon by ringer or saline solution (500cc /8h for 1 or 2 days) to evacuate the colon from fecal matter and for mechanical preparation of the colon, during this period all patients were kept nothing per mouth.

**Fig. 1:**
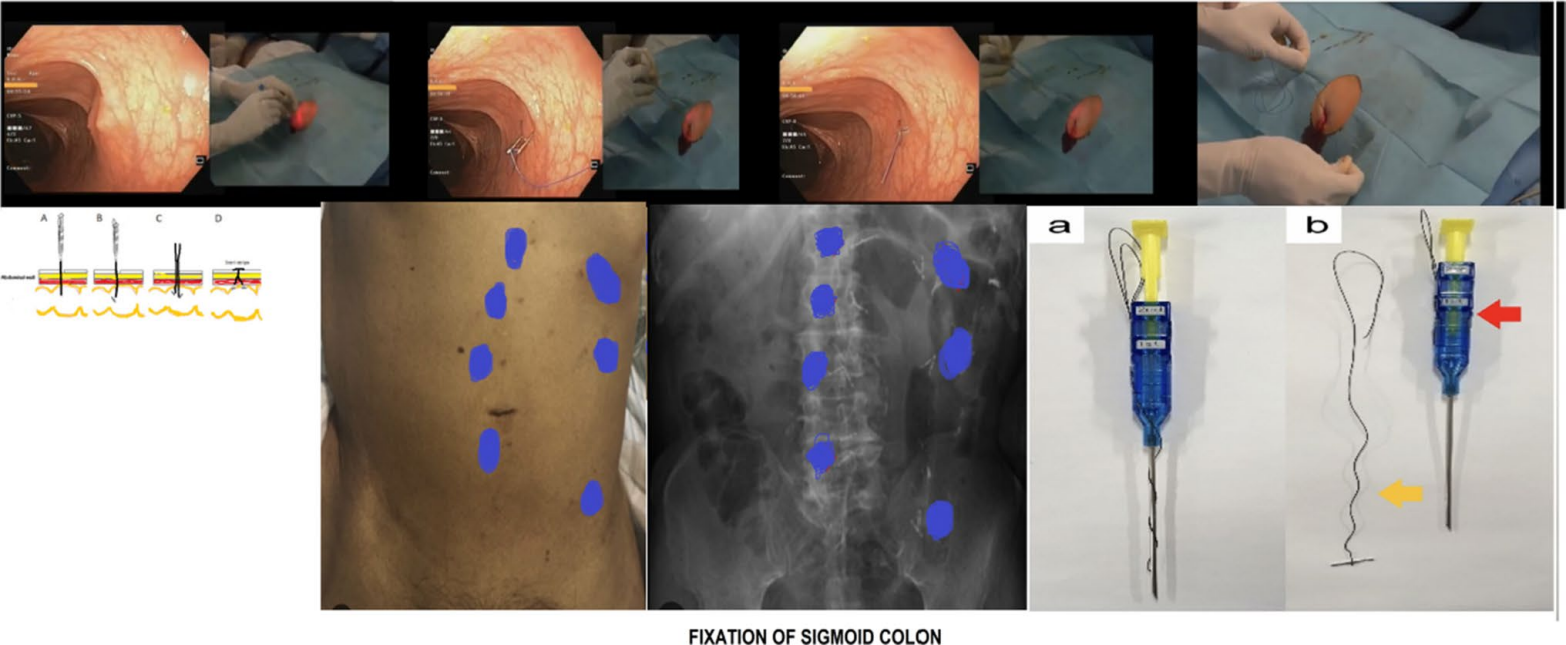
Fixation of sigmoid colon [11]

After that (on postoperative day 2 or 3 “POD 2 or 3”), patients were transferred again to operative theater (OR) for sigmoid colon fixation by endoscopy (as shown in Fig. 1):

1. The technique was performed by two doctors (one for endoscopic procedure and the other one for sigmoid fixation procedure).
2. The patients were sedated after intravenous preoperative ceftriaxone and metronidazole injection.
3. Then colonoscopy was performed in lithotomy position to locate the site of sigmoid colon fixation using radiological colonography by injection of radio-opaque dye via the endoscopy.
4. Then the tip of an artery forceps was externally positioned at anterior abdominal wall in the left lower quadrant to meet tip of colonoscopy or by trans-illumination against skin of anterior abdominal wall with indentation by index finger of surgeon’s hand. These points of fixation were arranged in circular manner, two parallel lines or even along the course of sigmoid with 1cm distance between each other. The pattern of points of fixation depended on the contour of patient’s abdomen, course and accessibility of sigmoid colon, and surgeon’s preference. In this study, the investigators used a two-shot anchor device (Olympus, Tokyo, Japan) that is used specifically for gastric fixation, to fix the sigmoid colon at anterior abdominal wall or Endo Close Device (Trocar Site Closure Device). The two-shot anchor device has two threads with T-shaped metal bar, each device is used for fixation at one point, each device has two buttons with a double, click top button, first thread with metal bar is released by pressing the first marked button with one press on top button for complete separation of the thread from the device, then the second thread is released by pressing the second marked button with another press on top button for complete separation from the device, then the two threads are pulled up against anterior abdominal wall then are tied together.
5. At this point, the skin was incised by scalpel about (2–3 mm puncture) then widening the puncture by artery forceps at level of subcutaneous tissue. A 2-shot anchor device (Olympus, Tokyo, Japan) was inserted to anchor and tether the sigmoid colon to anterior abdominal wall. We used a 20-gauge needle for further confirmation of site of fixation.
6. We performed a suction test to exclude the presence of any intervening organs between the sigmoid colon and anterior abdominal wall.
7. After that, the 2-shot anchor device was passed through the puncture to be entered inside the sigmoid colon and the thread with a metal T-bar was detached and pulled towards the anterior abdominal wall.
8. We repeated the previous steps at multiple points through multiple incisions (4–5 points of fixation).

On the following day before patient’s discharge, CT-colonography (virtual colonoscopy) was performed to confirm sigmoid fixation and avoid missing any leak.

Patients in surgical group underwent simple loop sigmoid colostomy proximal to site of obstruction in all patients in this group under general anesthesia by lower small exploratory midline incision, then deflating the sigmoid colon after untwisting, then simple loop sigmoid colostomy was done, then later after 6 weeks, all patients were subjected for surgical sigmoid fixation ( in 5 patients) or sigmoidectomy (in 4 patients due to large size colon and long mesentery of sigmoid colon or recurrence) after good colonic preparation either by open or laparoscopic approach according to patient general condition. For sigmoid fixation, closure of colostomy was done first by primary closure, and then extra-mural fixation of sigmoid colon was done to anterior abdominal wall by multiple interrupted simple sutures. However, sigmoidectomy was performed in cases of large size sigmoid colon with long mesentery and recurrent cases; resection anastomosis was done including site of colostomy by circular staplers.

### Statistical analysis

Analysis of data was performed by IBM computer using SPSS (statistical program for social science version 23): description of quantitative variables as median and range (IQR), Shapiro test of normality used to check the data distribution, description of qualitative variables as number and percentage, Chi-square test was used to compare qualitative variables between groups, Fisher exact test was used when one expected cell or more are less than 5, Mann–Whitney test was used. I considered the results statistically significant when the probability was less than 0.05 (*P* < 0.05). *P* value ≥ 0.05 was considered statistically insignificant [12].

## Results

**Fig. 2:**
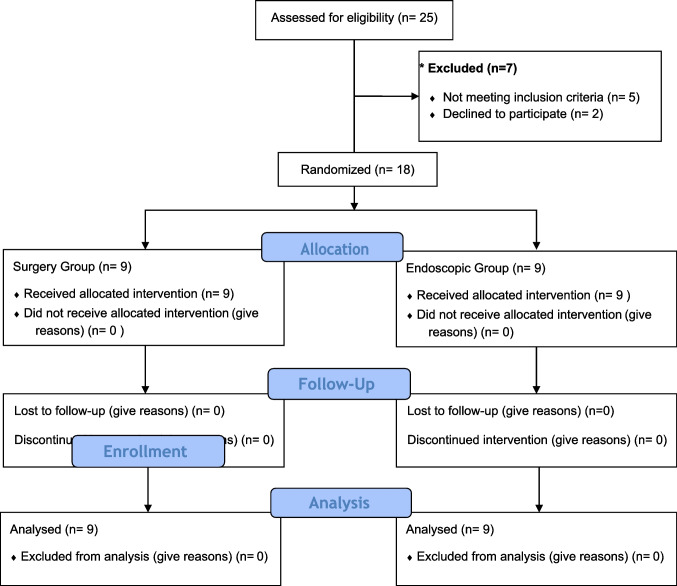
CONSORT flow diagram

Of 25 patients who presented with manifestations of acute sigmoid volvulus, eligible 18 patients were randomized into 2 groups: 9 patients in endoscopic group, and 9 patients in surgical group. Seven patients were excluded (in 5 patients: volvulus was resolved by deflating rectal tube and the remaining 2 patients refused to participate in the study) as shown in Fig. 2).

In this study, male patients constituted 33.3% (6/18). Six (33.3%) patients had past history of abdominal operations while 10 (44.4%) patients had recurrent volvulus. Regarding general condition, there were 12 patients with American Society of Anesthesiologists III class (66.70%) and 6 patients with American Society of Anesthesiologists IV class (33.30%) as shown in Table 1.

**Table 1:** Comparison between qualitative and quantitative variables of both groups

	Group A (endoscopy group ) *N*=9	Group B (surgical group ) *N*=9	Total column (*n*=18)	*P* value	Test
	Median (IQR)	Median (IQR)	Median (IQR)		
Age (years):	77 (70:80)	78 (72:80)	77.5 (70:80)	0.863	*Mann–Whitney ***u***
Sex	***N*** **(%)**	***N*** **(%)**	***N*** **(%)**	1	**Fisher exact test**
Male	3 (33.3)	3 (33.3)	6 (33.30)		
Female	6 (66.7)	6 (66.7)	12 (66.70)		
	Median (IQR)	Median (IQR)	Median (IQR)		
Body mass index (KG/M2)	29 (28:30)	30 (27:32)	29.5 (28:30)	0.863	*Mann–Whitney ***u***
American Society of Anesthesia classification	***N*** **(%)**	***N*** **(%)**	***N*** **(%)**	1	**Fisher exact test**
ASA III	6 (66.7)	6 (66.7)	**12 (66.70)**		
ASA IV	3 (33.3)	3(33.3)	**6 (33.30)**		
History of recurrent volvulus	***N*** **(%)**	***N*** **(%)**	***N*** **(%)**	1	**Fisher exact test**
Yes	4 (44.4)	4 (44.4)	**8 (44.40)**		
No	5 (55.6)	5 (55.6)	**10 (55.60)**		
Previous abdominal operation	***N*** **(%)**	***N*** **(%)**	***N*** **(%)**	1	**Fisher exact test**
Yes	3 (33.3)	3 (33.3)	**6 (33.30)**		
No	6 (66.7)	6 (66.7)	**12 (66.70)**		
Comorbidities	***N*** **(%)**	***N*** **(%)**	***N*** **(%)**		
Diabetes mellitus	3 (33.3)	2 (22.2)	**5 (27.80)**	1	**Fisher exact test**
HPN	3 (33.3)	2 (22.2)	**5 (27.80)**	1	**Fisher exact test**
Obesity	3 (33.3)	2 (22.2)	**5 (27.80)**	1	**Fisher exact test**
Ischemic heart disease	0 (0)	1 (11.1)	**1 (5.60)**	1	**Fisher exact test**
Patient with compensated chronic liver cirrhosis	0 (0)	1 (11.1)	**1 (5.60)**	1	**Fisher exact test**
Patient with chronic renal failure	0 (0)	1 (11.1)	**1 (5.60)**	1	**Fisher exact test**
Need for general anesthesia	***N*** **(%)**	***N*** **(%)**	***N*** **(%)**		
Yes	0 (0)	9 (100)	**9 (50)**	<0.001	**Fisher exact test**
No	9 (100)	0 (0)	**9 (50)**	<0.001	**Fisher exact test**
	Median (IQR)	Median (IQR))	Median (IQR))		
Procedure time in minutes	25 (20:30)	90 (65:100)	**42.5 (25:90)**	<0.001	*Mann–Whitney ***u***
Hospital stays in days	3 (2:3)	6 (5:7)	**4 (3:6)**	<0.001	*Mann–Whitney ***u***
Observation period post second procedure in days	8 (7:10)	8 (7:9)	**8 (7:10)**	0.863	*Mann–Whitney ***u***
Post-operative complications	***N*** **(%)**	***N*** **(%)**	***N*** **(%)**		
Chest infection	2 (22.2)	3 (33.3)	**5 (27.80)**	1	**Fisher exact test**
Skin necrosis	0 (0)	3 (33.3)	**3 (16.66)**	0.206	**Fisher exact test**
Peritonitis	0 (0)	3 (33.3)	**3 (16.66)**	0.206	**Fisher exact test**
Pelvic abscess	0 (0)	3 (33.3)	**3 (16.66)**	0.206	**Fisher exact test**
Small intestinal injury	0 (0)	1 (11.1)	**1 (5.60)**	1	**Fisher exact test**
Wound infection	0 (0)	3 (33.3)	**3 (16.66)**	0.206	**Fisher exact test**
Mortality	***N*** **(%)**	***N*** **(%)**	***N*** **(%)**		
Yes	0(0)	1(11.1)	**1 (5.60)**	1	**Fisher exact test**
No	9 (100)	8 (88.9)	17 (94.40)	1	**Fisher exact test**
Quality of life	***N*** **(%)**	***N*** **(%)**	***N*** **(%)**		
Excellent	8 (88.9)	3 (33.3)	11 (61.11)	0.05	**Fisher exact test**
Good	1 (11.1)	5 (55.6)	6 (33.33)	0.05	**Fisher exact test**
Poor	0 (0)	1 (11.1)	1 (5.55)	0.05	**Fisher exact test**

All patients in surgical group needed general anesthesia. Patients in both groups had comorbidities; there were 5 (27.80%) patients with diabetes mellitus, 5 (27.80%) patients with hypertension, 5 (27.80%) obese patients, one (5.60%) patient with ischemic heart disease, one (5.60%) patient with compensated chronic liver cirrhosis, and one (5.60%) patient with chronic renal failure. In both groups, there was no recurrence of volvulus. The reported complications were chest infection, skin necrosis, peritonitis, pelvic abscess, small intestinal injury, wound infection, and mortality with incidence of occurrence as follows: (5/18; 27.80%), (3/18; 16.66%), (3/18; 16.66%), (3/18; 16.66%), (1/18; 5.60%), (3/18; 16.66%), and (1/18; 5.60%) patient, respectively. During the 9-month follow-up period, endoscopic group demonstrated no cases of recurrence post-fixation as shown in Table 1.

In comparison between both groups regarding age, body mass index, length of hospital stay, procedure time, and observational time post procedure, there was a statistically significance difference regarding hospital stay and procedure time as shown in Table 1. Also, in comparison between both groups regarding sex, American Society of Anesthesiologists class, need of general anesthesia, mortality, history of recurrent volvulus, and previous abdominal operation, there was statistically significant difference regarding the need for general anesthesia as shown in Table 1. Also, there was no statistically significant difference regarding postoperative complications and co-morbidities as shown in Table 1**.** In surgical group, complications occurred after the second procedure; not after simple loop sigmoid colostomy. In endoscopic group, post-operative complications after second procedure were chest infection, skin necrosis, peritonitis, pelvic abscess, small intestinal injury, and wound infection with incidence of occurrence (2/9; 22.2%), (0/9; 0%), (0/9; 0%), (0/9; 0%), (0/9; 0%), and (0/9; 0%), respectively. Meanwhile, the incidence rates of this complications in surgical group were (3/9; 33.3%), (3/9; 33.3%), (3/9; 33.3%), (3/9; 33.3%), (1/9; 11.1%), and (3/9; 33.3%), respectively. There was no mortality in endoscopic group unlike the surgical group, as there was one mortality case.

Eight patients in the endoscopy group had an excellent quality of life, and one had good quality of life; unlike the surgical group, there were 3 patients with excellent quality of life, 5 patients with good quality of life, and 1 patient with poor quality of life, so there was statistically significant difference regarding quality of life among both groups as shown in Tables 1 and 2; the quality of life was evaluated after sigmoid fixation or excision and during 9-month follow up period by WHOQOL-BREF questionnaire that involved 26 items [13].

**Table 2:** Quality of life

Quality of life	Group A (endoscopy group) *N*=9	Group B (surgical group) *N*=9	*P* value	Relative risk	95th CI	
	*N* (%)	*N* (%)				
Excellent	8 (88.9)	0 (0)	<0.001	10	1.558	64.198
Good/poor	1 (11.1)	9 (100)				

## Discussion

As the incidence rate of acute sigmoid volvulus is high in elderly patients and is usually associated with serious and fatal complications if remains untreated, and also as the elderly patients usually have much co-morbidity, the least invasive maneuver to treat volvulus should be advised [14]. The endoscopic approach has both diagnostic and therapeutic advantages that make it a corner stone in managing volvulus [15].

In addition to its diagnostic and therapeutic advantages, the endoscopic approach is characterized with shorter operative time, shorter length of hospital stay, faster management, and no need for general anesthesia. For these advantages, the endoscopy has the upper hand in managing volvulus, especially in elderly patients with much co-morbidity [16].

In this study, we included patients with body mass index below 35 as the higher body mass index is usually associated with failure of endoscopic fixation because of thick and fatty anterior abdominal wall that makes it difficult for two-shot anchor device to reach sigmoid colon lumen, so the thinner the patient, the higher success rate.

In endoscopic group, after endoscopic untwisting of volvulus, patients were subjected to fixation within 1 or 2 days after colonic preparation while in surgical group, patients had been firstly subjected to colostomy then after at least 6 weeks, they underwent the definitive surgery; hence, the quality of life was better in the endoscopic group.

In this study, there was no need for general anesthesia in the endoscopic group, so the endoscopic approach seems suitable for patients with American Society of Anesthesiologists III and IV classes and elderly patients with many comorbidities, as well.

Also, endoscopic fixation is feasible in those patients with a past history of recurrent volvulus and/or past history of previous abdominal operation as in this study, unlike the traditional surgery for sigmoid volvulus as when it is performed in patients with a past history of abdominal surgery, it may be associated with a high incidence of lethal complications such as intestinal injury because of the existing adhesions.

In this study, the complications included chest infection, skin necrosis, peritonitis, pelvic abscess, small intestinal injury, and wound infection. In the endoscopic group, only 2 patients developed fever because of chest infection. Other complications did not occur in the endoscopic group as the site of fixation was only 2mm in length, so the incidence rate of wound infection was extremely rare. In this study, various complications such as intestinal injury, fecal fistula, pelvic abscess, and peritonitis were not seen in endoscopic group because all procedure’s steps were performed under radiological guidance. However, in the surgical group, there were 3 patients with chest infection, 3 patients with peritonitis (due to fecal fistula, anastomotic leak, and small intestinal injury), 3 patients with pelvic abscess (due to anastomotic leak and small intestinal injury), 1 patient with small intestinal injury (this occurred in patient with history of previous abdominal surgery due to extensive adhesions), 3 patients with wound infection (due to fecal fistula), and 3 patients with skin necrosis at colostomy site (due to fecal fistula). Mortality has occurred in one patient in surgical group due to fecal fistula, peritonitis, and septic shock. In surgical group patients, the complications occurred 6 weeks later after sigmoid fixation or excision.

In comparison to the present study, Imakita et al. included 8 patients with American Society of Anesthesiologists classes > 3 and excluded patients with intestinal ischemia and necrosis. Imakita et al. performed colonoscopy assisted percutaneous sigmoidopexy in 8 patients, with median age of 72.5 years. Their results showed that median procedure time was 72.5 min; median time for fixation was 16 min; the only perioperative adverse event elicited was subcutaneous emphysema in one patient; no patients developed peritonitis, small intestinal injury, peritoneal collection, wound infection, nor intestinal obstruction. In the previously mentioned study, no recurrence occurred during the 1-year follow up period, while 3 patients died due to aspiration pneumonia after 4 months (not related to the procedure). So our study is in consistence with Imakita et al. that there was no recurrence after endoscopic fixation of sigmoid colon with no other complications related to the procedure [17].

We used WHOQOL-BREF questionnaire that has 26 items to assess the quality of life. This questionnaire has four domains included which are physical health domain (7 items), psychological health domain (6 items), social relationship domain (3 items), and environmental health domain (8 items); it also has quality of life and general health item. Each separate item is scored from 1 to 5 on a response scale. The scale is then stipulated as a five point ordinal scale. The score is then changed linearly to a 0–100 scale. The physical health domain includes items on mobility, daily activities, functional capacity, energy, pain, and sleep. The psychological domain includes items on self-image, negative thoughts, positive attitude, self-esteem, mentality, learning ability, memory concentration, religion, and mental status. The social relationship domain includes items on personal relationship, social support, and sex life. The environmental health includes items on financial resources, safety, health and social services, living physical environment, opportunities to acquire new skills and knowledge, recreation, general environment (noise, air pollution, etc.), and transportation. The score below 50 means poor quality of life, score between 50 and 75 means good quality of life, and score more than 75 means excellent or high quality of life. The quality of life was assessed 9 months after sigmoid fixation in endoscopic group or sigmoid fixation or excision in surgical group.

This study has some limitations, the small sample size that may not give powerful statistical conclusions. The small sample size was due to low incidence rate of sigmoid volvulus. Inclusion of only elderly patients with American Society of Anesthesiologists III and IV classes, and exclusion of patients < 60 years old and patients who responded to deflating rectal tube are other limitations of this study (those responders to deflating rectal tube were excluded and managed later by sigmoid fixation either by surgery or endoscopy; or sigmoid excision; or follow up according to the patient’s general condition or patient’s choice, as this study was mainly directed towards high risk surgical elderly patients who did not respond to rectal deflating tube to avoid morbidities and mortality associated with untwisting the sigmoid colon. More large studies should be performed to include different categories of the patients in order to prove the efficacy and safety of endoscopic sigmoid fixation). Many other trials should be done in the future for all patients with sigmoid volvulus as endoscopic sigmoid fixation may be safe and effective in all patients. The strength of the present study is being a randomized controlled trial that compared endoscopic intervention on one hand with the surgical intervention on the other hand. Also, it proved the efficacy and safety of the endoscopic fixation of sigmoid colon in elderly high risk surgical patients. Moreover, it shortened the wait period before sigmoid fixation or excision (median waiting time was 6 weeks**).**

## Conclusion

Endoscopic management of acute sigmoid volvulus is effective and safe in elderly high risk surgical patients (either in managing the intestinal obstruction caused by volvulus or in definitive treatment of volvulus).

## Data Availability

All data generated during this study are included in this published article and its supplementary information files. Further, minor datasets are available from the corresponding author on reasonable request.
